# Tuning Rex rules HTLV-1 pathogenesis

**DOI:** 10.3389/fimmu.2022.959962

**Published:** 2022-09-16

**Authors:** Kazumi Nakano, Toshiki Watanabe

**Affiliations:** ^1^ Department of Computational Biology and Medical Sciences, Graduate School of Frontier Sciences, The University of Tokyo, Tokyo, Japan; ^2^ Department of Practical Management of Medical Information, Graduate School of Medicine, St. Marianna University, Kawasaki, Japan

**Keywords:** HTLV-1 Rex, HTLV-1 Tax, HTLV-1 Hbz, ATL, HIV-1 Rev, AIDS, viral replication, latent infection

## Abstract

HTLV-1 is an oncovirus causing ATL and other inflammatory diseases such as HAM/TSP and HU in about 5% of infected individuals. It is also known that HTLV-1-infected cells maintain a disease-free, immortalized, latent state throughout the lifetimes of about 95% of infected individuals. We believe that the stable maintenance of disease-free infected cells in the carrier is an intrinsic characteristic of HTLV-1 that has been acquired during its evolution in the human life cycle. We speculate that the pathogenesis of the virus is ruled by the orchestrated functions of viral proteins. In particular, the regulation of Rex, the conductor of viral replication rate, is expected to be closely related to the viral program in the early active viral replication followed by the stable latency in HTLV-1 infected T cells. HTLV-1 and HIV-1 belong to the family *Retroviridae* and share the same tropism, e.g., human CD4^+^ T cells. These viruses show significant similarities in the viral genomic structure and the molecular mechanism of the replication cycle. However, HTLV-1 and HIV-1 infected T cells show different phenotypes, especially in the level of virion production. We speculate that how the activity of HTLV-1 Rex and its counterpart HIV-1 Rev are regulated may be closely related to the properties of respective infected T cells. In this review, we compare various pathological aspects of HTLV-1 and HIV-1. In particular, we investigated the presence or absence of a virally encoded “regulatory valve” for HTLV-1 Rex or HIV-1 Rev to explore its importance in the regulation of viral particle production in infected T cells. Finally, wereaffirm Rex as the key conductor for viral replication and viral pathogenesis based on our recent study on the novel functional aspects of Rex. Since the activity of Rex is closely related to the viral replication rate, we hypothesize that the “regulatory valve” on the Rex activity may have been selectively evolved to achieve the “scenario” with early viral particle production and the subsequent long, stable deep latency in HTLV-1 infected cells.

## Introduction

More than 40 years have passed since the discovery of HTLV-1, and much knowledge has been accumulated in the analysis of its characteristics as an oncovirus, especially through the functional analysis of viral functional proteins such as Tax and HBZ, as well as through the tracking of mutational evolution of infected cells (1–3). Although HTLV-1 does cause ATL (adult-T cell leukemia/lymphoma, about 5% of carriers in Japan) (4–6), HAM/TSP (HTLV-1 associated myelopathy/tropical spastic paraparesis, about 0.3% of carriers in Japan) (7) and HU (HTLV-1 uveitis, about 0.1% of carriers in Japan) (8), it does not cause diseases in rest of about 95% of infected individuals for the rest of their lives (1, 5, 9). Most infected cells are expected to remain in a stable latent state (deep latency=transcriptional latency) in the carrier body (9). It is speculated that neither active proliferation of infected T cells nor virion production for *de novo* infection occurs in asymptomatic carriers since the proviral load (PVL=% proviral DNA carrying T cells in PBMCs) is kept under 4% in most asymptomatic carriers (6). HTLV-1 is thought to have co-evolved with the human life cycle over at least tens of thousands of years since when STLV-1 (simian T-cell leukemia virus type-I), which was widespread in the old-world monkeys, infected humans. The reason why it was able to spread around the world with human migration over a long period without being eliminated from human life is thought to be that HTLV-1 was essentially a virus that did not kill human hosts. We, therefore, believe that the long-term maintenance of disease-free infected cells in the carrier body is a low-risk transmission strategy of this virus. Development of life-threatening ATL, i.e., tumorigenesis of infected cells, is therefore considered as a deviation from the viral program.

HIV-1, which belongs to the same family *Retroviridae* as HTLV-1, has many similarities in the tropism, genomic structure, and viral replication cycle with those of HTLV-1 (10). Unlike HTLV-1 infected cells, it is known that HIV-1 infected cells sustain viral production at a low level even during latency (=shallow latency) and resume active viral production (reactivation) by antigen stimulation and cytokine signaling without the antiretroviral therapy (ART) (11). The viral particle production is continued until the death of the host cell. *De novo* infection of functional T cells leads to depletion thus nearly 100% of infected individuals eventually develop acquired immunodeficiency syndrome (AIDS) if not treated (12). The difference in virulence between HTLV-1 and HIV-1 seems to be closely related to the viral replication capacity in acute and latent infection. Since HTLV-1 Rex and its HIV-1 counterpart, Rev, play a central role in the positive regulation of viral replication rate, we hypothesized that regulation of HTLV-1 Rex and HIV-1 Rev is related to the virulence of HTLV-1 and HIV-1, respectively. In this context, we investigated the mechanism of the *cis*-acting “regulatory valve” for Rex and Rev in HTLV-1 and HIV-1, respectively. Moreover, we explored if the presence or absence of such a “regulatory valve” for HTLV-1 Rex and HIV-1 Rev is indeed reflected in the viral production rate of infected cells and the pathogenesis of each virus. Finally, our recent study, which demonstrated that Rex intervenes in the various cellular pathways to achieve its function, proposed the possibility that Rex is an effective target of regulation for the virus to control the overall viral replication efficiency in the infected cells. Thus, the regulatory mechanism of Rex may be selectively equipped during the evolution of HTLV-1 to achieve the “scenario” with early viral production followed by a long, disease-free latency of infected cells.

## Evolution of HTLV-1

### Transmission from monkeys to humans

The origin of HTLV-1 is the cross-species transmission of STLV-1 (simian T-cell leukemia virus type-I) from non-human primates (NHPs) to humans in Central Africa. Subsequently, it spread throughout the world with the geographic movement of people. Currently, seven types of HTLV-1a-g have been identified, and HTLV-1a has an additional eight subtypes, A-F and Senegalese. There is little genomic variation among the HTLV-1 types, indicating that the virus has spread to the human world *via* transmission and proliferation of infected cells rather than through novel infection by viral particles, which generally increases the frequency of recombination and point mutations. According to molecular clock calculations, STLV-1 is predicted to have infected humans and diverged to HTLV-1 93000+/-8000 years ago (13). Among seven types of HTLV-1, HTLV-1c is highly varied, and this strain is estimated to be confined and isolated in Melanesian Islands and Australia at a relatively early stage, about 40,000~60,000 years ago (13, 14), while HTLV-1a,b,d, and e diverged 27300+/-8200 years ago (15). Thus, HTLV-1 has evolved over tens of thousands of years in humans without being eliminated from the human life cycle.

### HTLV-1 survival strategy in the human life cycle

**Figure 1:**
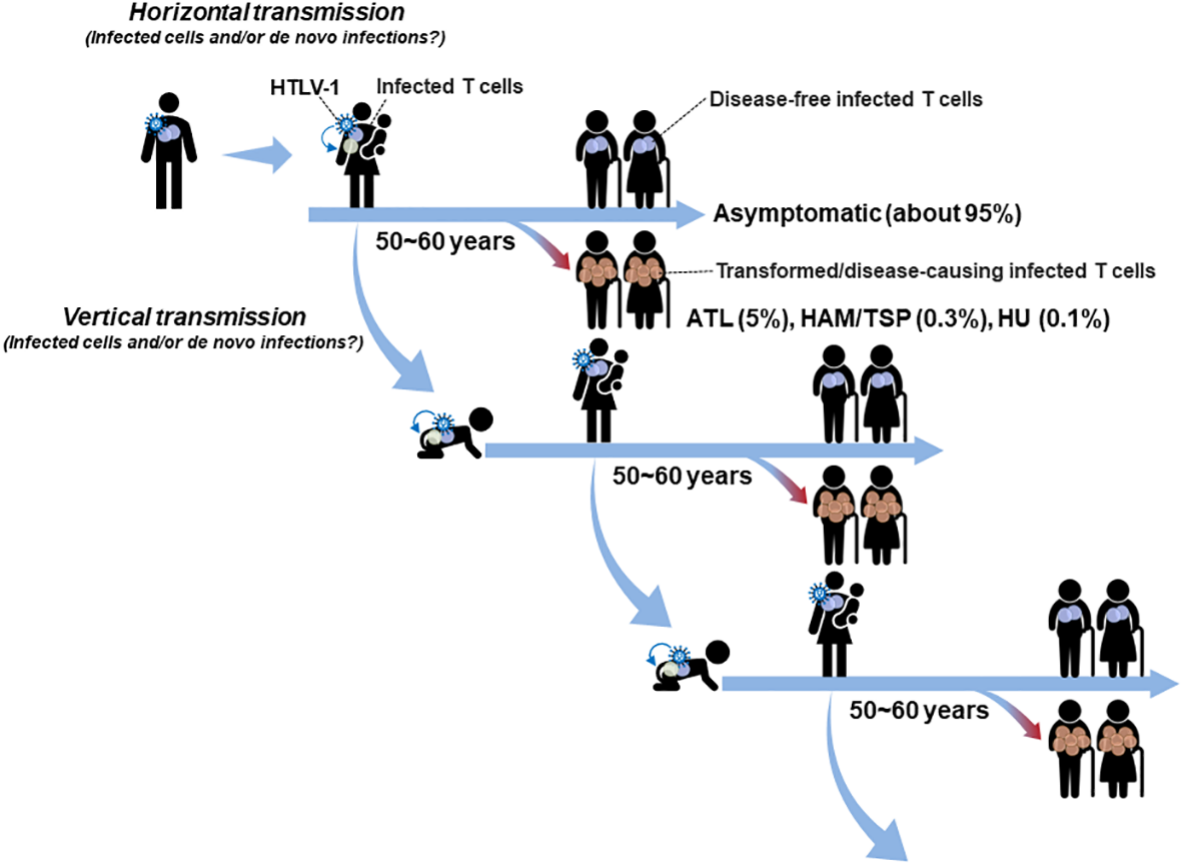
Mode of HTLV-1 transmission in the human life cycle: HTLV-1 is mainly transmitted by vertical mother-to-child transmission *via* breast milk or by horizontal sexual transmission and blood transfusion. Both modes of transmission, i.e., the transmission of infected T cells and/or infectious virions, are possible. Although HTLV-1 causes ATL, HAM/TSP, and HU, only about 5% of carriers develop diseases (mostly ATL) and about 95% of carriers remain asymptomatic, in which infected cells maintain a disease-free latent state for their lifetimes. Especially, HTLV-1 infected cells transferred from the carrier mother as well as *de novo* established in the infant are expected to quickly be immortalized and remain in a stable latent state for decades beyond the age to transmit the virus to the next generation.

HTLV-1 is transmitted vertically through breast milk or horizontally through sexual transmission or blood transfusion (**Figure 1**). Although the mode of transmission has not been clarified yet in both vertical and horizontal transmissions, both possibilities, i.e., HTLV-1 infected cell transmission and/or infectious virion transmission have been proposed by *in vitro* infection experiments and *in vivo* animal model (16). As summarized in the review by Miura et al. (17), reactivations of the sense-strand viral gene expression by *ex vivo* culture of PBMCs from HTLV-1 infected individuals are often observed. Therefore, it is also possible that infected T cells, which are latent in the donor body, may be reactivated at the time of transmission in the acceptor body.

A recent analysis of blood donors in Japan, which is one of the endemic areas of HTLV-1 infection, showed that the proportion of horizontally transmitted cases is unexpectedly high (approximately 4,000 cases/year, with a peak age of 50-59 years for women and 60-69 years for men) and that 77% of cases are transmitted from men to women (18). The average age of onset of ATL in Japan is 67 years old (5), and the peak age of onset of HAM/TSP is in the 40~50 age group (7). Further epidemiologic analyses are required to clarify the proportion of vertical and horizontal infection, respectively, involved in the development of HTLV-1-associated disease. At least based on the reports showing that the peak age of horizontal transmission is higher than 50 years old for men and women, it is likely that the disease-causing infection for the most ATL and a part of HAM/TSP and HU occurs through vertical transmission (16, 19). Indeed, it is widely accepted that HAM/TSP and HU are often observed to be developed over a period of months to years due to horizontal transmission such as blood transfusions (8, 20–24). In addition, intergenerational (vertical mother-to-child) transmission may be an important evolutional factor for HTLV-1 in the continuous presence of this virus in the human life cycle (**Figure 1**). Although HTLV-1 is the human oncovirus that causes several diseases including ATL, infected individuals develop ATL at low rates same as other oncoviruses (25, 26). The incidence of ATL, HAM/TSP, and HU is less than 5% of HTLV-1 carriers, and about 95% of carriers remain asymptomatic for their lifetimes. After the transmission, immortalized infected cells remain in a disease-free latent state in most cases until he/she is old enough to transmit the virus to the next generation (**Figure 1**).

### Unanswered question: how do most of HTLV-1 infected cells remain disease-free?

We still do not know the viral mechanism, which allows HTLV-1 to establish a stable disease-free latency for decades. We hypothesize that HTLV-1 has an inherent program that makes its accessory proteins function in a coordinated or antagonistic manner to establish an infected cell of such characteristics. HTLV-1 was discovered as a novel human retrovirus in 1980~81 (27, 28). In the following year, it was identified as the virus causing ATL (29, 30), followed by the elucidation of its genomic structure (31). Subsequently, by the early 2000s, the function of accessory proteins encoded by the pX region and their effects on host T cell pathways were vigorously analyzed, and the molecular mechanisms of viral replication in infected T cells were elucidated (32–35). Those studies contributed to clarifying a wide scope of HTLV-1 molecular mechanism in viral replication, yet not fully understanding the whole landscape of HTLV-1 infected cells from the early phase to the latent state. From the late 2000s, the research interest was shifted to the aspect of HTLV-1 as an oncovirus. In particular, the importance of Tax and Hbz in tumorigenesis of infected cells was highlighted. There are many reports demonstrating the transforming activity of Tax and Hbz *in vitro* and *in vivo* (36–45). However, given that most infected individuals do not develop ATL, HTLV-1 may have a mechanism to downregulate the transforming activities of Tax and Hbz. It is well-known that Tax expression is turned-off in the early phase of infection, thus the latently infected cells can escape from the host immune response (46). On the other hand, Hbz is not effectively targeted by HTLV-1-specific cytotoxic T cells, allowing Hbz to be continuously expressed in latently infected T cells (47). We do not have a clear answer to why most infected individuals do not develop ATL even though Hbz is expressed in almost all infected cells of HTLV-1 infected individuals. The regulatory mechanism among viral proteins is an important field of research, which is remained to be elucidated. In 2007 and 2008, reports were successively published on the regulation of viral gene expression by the mutual regulation mechanism of Rex and p30II (48, 49). More recently, Philip et al. reported that Hbz negatively regulated the function of Rex (50).

We have been focusing on the importance of Rex in the HTLV-1 life cycle. Although Rex is thought to regulate the timing of viral replication and latency transition through its function of transporting viral RNA (51, 52), how it fulfills this function is largely unknown. Therefore, we believe it is important to elucidate novel functional aspects of Rex that remain mostly to be explored. Rex transports viral unspliced/partially spliced mRNAs out of the nucleus. We previously reported that Rex suppresses the host mRNA quality control mechanism, NMD (nonsense-mediated mRNA decay), and stabilizes HTLV-1 RNA, a target of NMD (53, 54). However, it is still not known how it avoids splicing that occurs simultaneously with transcription. It is also not elucidated if Rex increases the selective translation efficiency of viral mRNAs.

Overall, to clarify the nature of HTLV-1, it is essential to understand the events occurring in realistic infected cells where multiple viral accessory proteins co-exist. Active viral replication occurs in the early phase of infection, followed by immortalization and the transition to the latent state. The detailed mechanism to achieve such a “scenario” has not been clarified, yet. We speculate that the viral mechanism to maintain an appropriate power balance among the accessory proteins such as Tax, Rex, and Hbz is essential for the “scenario”, yet our knowledge of the such aspect of the virus is far from enough. The interplay among viral accessory proteins should be further investigated to understand the viral characteristics and pathogenesis of HTLV-1.

## Similarities and differences between HTLV-1 and HIV-1

**Figure 2:**
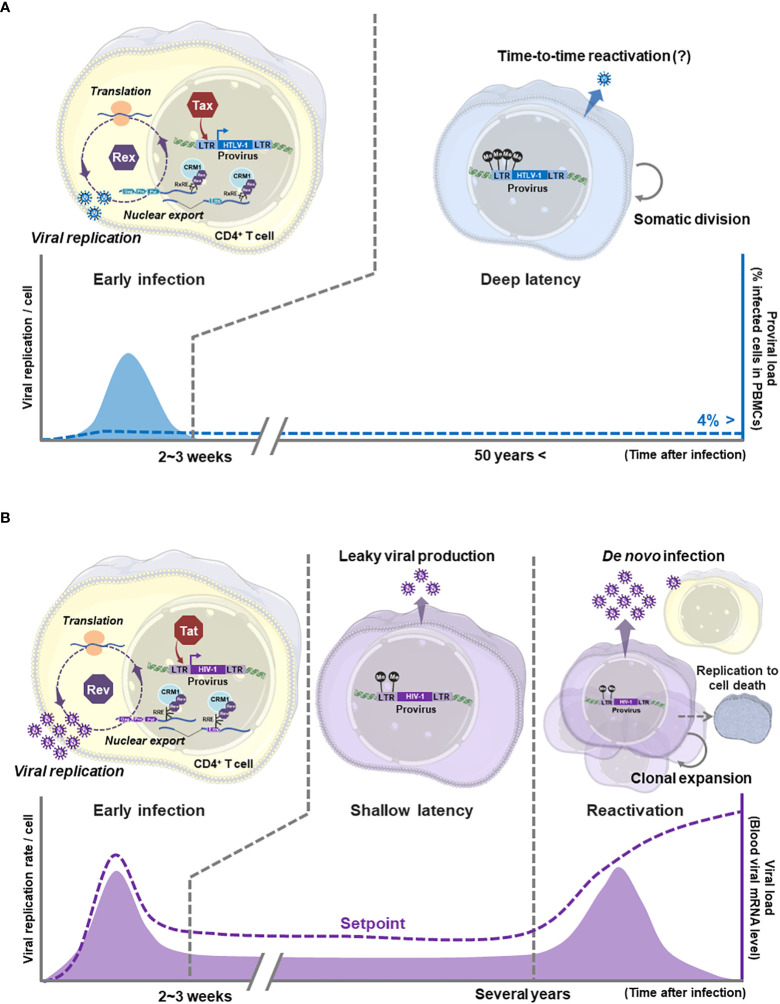
Comparison of HTLV-1 and HIV-1 viral replication modes: **(A)** In the early state of HTLV-1 infection to T cells, Tax and Rex are expressed from the provirus to start viral gene expression. Tax activates the viral promoter 5′LTR (as L.545) and enhances transcription of unspliced/singly spliced mRNAs encoding viral structural proteins. Rex nuclear exports those unstable, not-completely spliced viral RNAs *via* the RxRE/CRM1 dependent mechanism to enhance the translation of viral structural proteins, thus the production of virions. In HTLV-1 infected cells, such active virion replication is expected to be brought to the end within a few weeks. Then HTLV-1 infected cells transit to the latency with immortalization, although the molecular mechanism of immortalization has not been clarified. The immortalized HTLV-1 infected T cell maintains the deep latency (transcriptional latency) without active viral gene expression for decades. Somatic infected-cell division and temporal virion production may occur to maintain the infected cell number in asymptomatic carriers. The ProVL is the % of provirus-carrying inactive infected cells in PBMCs of an infected individual (blue dashed line). **(B)** HIV-1 also undergoes active viral particle replication through Tat-mediated activation of 5′LTR (as L.545) and nuclear export of unspliced/singly spliced viral mRNAs by Rev/RRE/CRM1 dependent mechanism. The active viral replication in infected cells and *de novo* infection *via* viral particles quickly increase the viral load (VL: the number of HIV-1 mRNAs in a carrier body=the blood level of virus particles, purple dashed line) to the peak level within a few weeks in the acute infection phase. After the acute infection, the infected cells are quickly eliminated leaving a small number of long-lived memory CD4^+^ T cells as the reservoir. The reservoir cells are expected to produce virions at a low level during the (shallow) latent infection, therefore the VL is reduced to the setpoint (no disease-inducing level), but never becomes undetectable level in untreated carriers. Without treatment, the reservoir cells are reactivated within a few years and clonally expanded by immunological stimuli. Reactivated cells produce a vast number of viral particles, resulting in an explosive increase in the number of newly infected T cells and the VL. The viral particle production is continued until the host cells die, thus the number of functional CD4^+^ T cells is depleted to cause the onset of AIDS. Generated using Servier Medical Art, provided by Servier, licensed under a Creative Commons Attribution 3.0 unported license.

HTLV-1 belongs to the family *Retroviridae* and the genus *Deltaretrovirus*. HIV-1 also belongs to the family *Retroviridae* and the genus *Lentivirus*. As retrovirus, HTLV-1 and HIV-1 have a single-strand sense RNA as the viral genome. After entering human T cells, the genomic RNA is reverse transcribed and further transformed into double-stranded DNA, which is rapidly integrated into the human genomic DNA as a provirus. HTLV-1 and HIV-1 have much in common in terms of the genome structure, the infection topology, the mode of infection, and the viral replication pathways (10, 55–57). On the contrary, HTLV-1 and HIV-1 show significant differences in the mode of infection and pathogenesis (**Figure 2**). To understand the characteristics of HTLV-1, comparisons with HIV-1 could be a useful tool, since more knowledge, especially in molecular virology including structural details of viral proteins, has been accumulated (58–66) than HTLV-1.

### Receptors and tropisms

The major population of both HTLV-1 and HIV-1 infected cells in infected individuals is CD4^+^ T cells. The tropism of HIV-1 in CD4^+^ T cell is relevant since it enters *via* interaction between CD4 on the host cell and viral Env-gp120 with CCR5 or CXCR4 as co-receptors (67). In contrast, GLUT1, a ubiquitous glucose transporter, and NRP-1, a glycoprotein more specific to T cells and dendritic cells (DCs), have been identified as the cellular receptor for HTLV-1 with heparan-sulfate proteoglycan as a cofactor essential for viral attachment (68). Although GLUT1 is known to be expressed in a wide variety of cell types, the expression level in the resting T cell is kept low, while increased by T cell activation (68), which may be related to the more specific tropism of HTLV-1 than the receptor distribution. Yet, the cause of the strong HTLV-1 tropism in CD4^+^ T cells in infected cells in asymptomatic carriers and disease-causing cells in HAM/TSP patients and ATL patients has not been fully elucidated.

### Regulation of viral gene expression during early infection

#### The LTR activation by HTLV-1 tax and HIV-1 tat

In both HTLV-1 and HIV-1, after the entry to the host cell, the viral genomic RNA is reverse-transcribed and converted to a double-stranded DNA to be integrated into the host genome as the provirus (10). It has been well defined that the HIV-1 provirus is tended to be integrated downstream of an actively-transcribed gene, which is regulated by the host nuclear architecture (69). Less preference for the integration site of HTLV-1 provirus was reported firstly in HeLa cells, which were experimentally infected with HTLV-1 (70). Gillet et al. demonstrated for the first time in the HTLV-1 infected individuals that a significantly higher frequency of proviral integration in transcriptionally silenced genes was observed in asymptomatic carriers (71). More recently, a significantly higher preference for actively-transcribed genes was observed in infected cells in HAM/TSP patients compared with asymptomatic carriers (72), indicating an implication between the HTLV-1 proviral integration site and the risk of disease onset.

In early infection, the viral gene expression occurs *via* activation of the 5´ LTR (long terminal repeat) by Tax for HTLV-1 (**Figure 2A**) and Tat for HIV-1 (**Figure 2B**), respectively. HTLV-1 Tax binds to TREs (Tax responsive elements) in the LTR *via* a cellular transcription factor CREB (cAMP response element binding protein). The Tax-CREB complex then recruits strong cellular co-activators such as p300 (E1A binding protein p300), CBP (CREB binding protein), and PCAF (P300/CBP-associated factor) to activate the LTR (35, 73). On the other hand, HIV-1 Tat directly binds to the HIV-1 LTR at the TAR (the trans-activating response) element. The HIV-1 LTR, which is embedded between two nucleosomes (nuc-0 and nuc-1), contains the NF-κB and the SP-1 binding sites to recruit these transcription factors. Thus mechanically, the HIV-1 LTR is always activated for transcription initiation. However, further elongation does not occur without Tat interaction with TAR because the RNA polymerase II (RNAPII) is stalled at nuc-1 without Tat. The binding of Tat on TAR recruits a transcription elongation factor P-TEFb, which directly activates RNAPII to resume elongation of viral transcripts by overcoming its stalling at nuc-1. The activity of Tat is also regulated by a series of cellular histone acetyltransferases (HATs), such as PCAF, p300/CBP, and hGCN5 (74).

#### Post-transcriptional regulation by HTLV-1 Rex and HIV-1 Rev

From the provirus of HTLV-1 and HIV-1, the doubly spliced mRNAs encoding accessory proteins, the singly spliced mRNA encoding Env, and the unspliced mRNA encoding Gag/Pro/Pol are transcribed from the provirus. At first, depending on the host cellular pathway, the doubly spliced *Tax/Rex* mRNA for HTLV-1 and *Tat* and *Rev* mRNAs for HIV-1 are transcribed. Then HTLV-1 Tax and HIV-1 Tat activate the 5´ LTR to enhance the transcription of unspliced/singly spliced viral mRNAs. For retroviruses, the nuclear export of those incompletely spliced viral mRNAs is essential for replication. It is noteworthy that the molecular mechanism of that nuclear export is different between complex retroviruses and simple retroviruses. Complex retroviruses, such as HTLV-1 and HIV-1, encode several accessory proteins including the viral RNA binding protein. Rex in HTLV-1 and Rev in HIV-1 are the accessory proteins playing the central role in the nuclear export of viral unspliced/singly spliced mRNAs. HTLV-1 Rex and HIV-1 Rev bind specifically to the Rex responsive element (RxRE) in the 3′ LTR (**Figure 2A**) and the Rev responsive element (RRE) in the Env coding region of the viral RNA (**Figure 2B**), respectively. Then, Rex and Rev themselves are transported by the cellular nuclear export adaptor protein CRM1, thereby promoting selective nuclear export of viral RNAs (**Figure 2**). Such active transport of unspliced/singly spliced viral mRNAs by HTLV-1 Rex and HIV-1 Rev enhances cytoplasmic accumulation and translation of viral structural proteins for viral particle replication (75, 76). HTLV-1 Rex and HIV-1 Rev return to the nucleus *via* the common host nuclear localization protein importing-β to repeat the nuclear export (56, 57, 77). On the other hand, simple retroviruses, such as MPMV (Mason-Pfizer monkey virus), do not have any accessory proteins. Instead, they have the RNA motif called the constitutive transport element (CTE) within an intronic region of the viral structural transcripts (77). The CTE-containing viral mRNA is nuclear-exported by direct binding of a host nuclear-export adaptor NXF1/Tap (nuclear RNA export factor 1/transporter associated with antigen processing) to CTE (78–80). NXF1 is also responsible for the modification and nuclear export of cellular mRNAs (81). Therefore the NXF1/CTE dependent pathway, which is a cellular pathway utilized by simple retroviruses, is distinguished from the CRM1-dependent Rex/RxRE and Rev/RRE pathways, which is a specific pathways by complex retroviruses (82). Indeed, HIV-1 Rev suppresses the cellular mRNA nuclear-export level by inhibiting recruitment of the transcription/export (TREX) complex with NXF1/Tap to the CAP structure of a cellular mRNA (83), probably to enhance the specific nuclear-export of viral transcripts. Recently, it was demonstrated that MLV (murine leukemia virus), a simple retrovirus, utilizes not only the NXF1/CTE dependent pathway for nuclear export of unspliced viral mRNA to be translated but also the CRM1 dependent pathway for nuclear export of unspliced genomic RNA for packaging (84). Each complex and simple retroviruses may have developed to hijack the host cellular mRNA transport machinery *via* a cellular canonical system (i.e., NXF1/Tap dependent) and/or the virus-specific system (i.e., CRM1 dependent) to optimize the viral gene expression level beneficial for replication.

**Figure 3:**
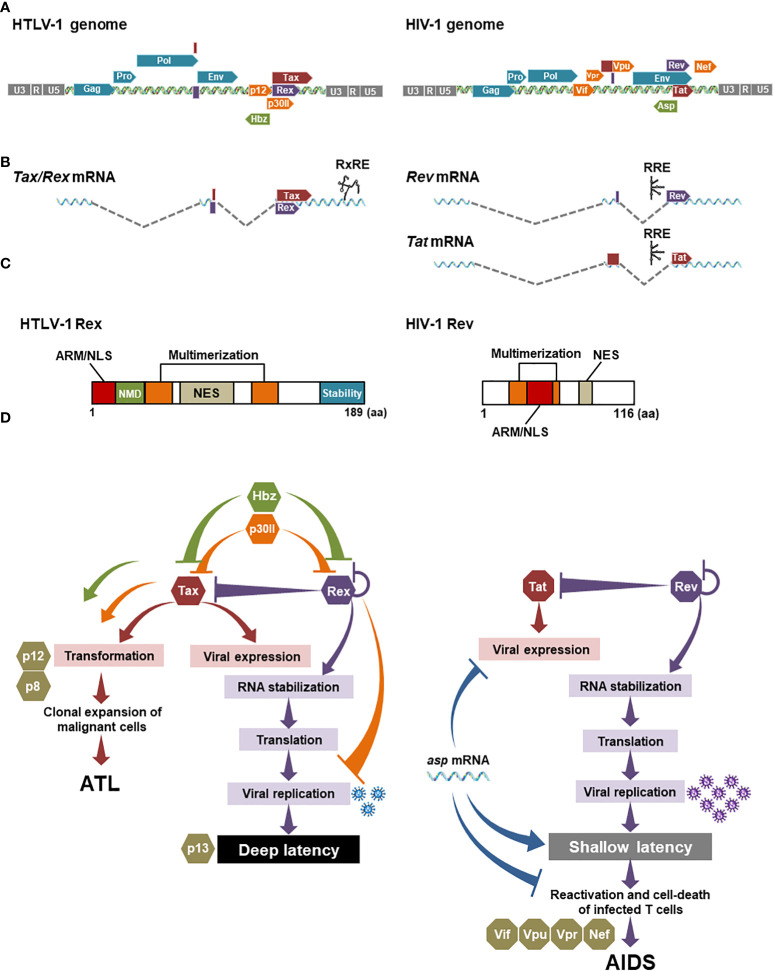
Comparison between HTLV-1 Rex and HIV-1 Rev: **(A)** The genomic structures of HTLV-1 and HIV-1 are similar, with structural proteins encoded on the 5′ side and viral accessory proteins including Rex and Rev on the 3′ side. **(B)** Of the several transcriptional variants transcribed from provirus, both Rex and Rev are encoded on the doubly spliced mRNAs. Importantly, HTLV-1 Rex and Tax are encoded on a single mRNA in different reading frames. HIV-1 Rev and Tat are encoded on separated mRNAs. **(C)** The primary amino acid structures of HTLV-1 Rex and HIV-1 Rev have common functional domains such as NES that binds CRM1, and NLS that binds importin-β, overlapping with the RNA-binding domain (ARM, arginine-rich motif), and two multimerization domains. Rex has a C-terminal stability domain and can remain within the cell longer than Tax. In addition, the region aa22-57 is involved in the inhibition of the host cell’s nonsense-mediated mRNA decay (NMD) (54). There is no clear stabilization domain reported so far in HIV-1 Rev. **(D)** In HTLV-1 and HIV-1 infected cells, Tax and Rex, and Tat and Rev are responsible for 5′LTR (as L.545) activation and viral mRNA stabilization, respectively. Rex functions to regulate the expression levels of self and Tax. Hbz negatively-regulates Tax and Rex activities in the early infected cells. P30II also suppresses Tax and Rex activities by sequestering *Tax/Rex* mRNA in the nucleolus. Tax, Hbz, and p30II are also known to be involved in the malignant transformation of infected T cells. P12 and its cleaved form p8 are not directly involved in the regulation of the viral replication cycle but regulate the proliferation of infected cells *via* the NFAT pathway. In HIV-1, Tat and Rev also play a central role in the viral replication cycle. Other accessory proteins such as Vif, Vpu, and Vpr influence infected cell phenotypes and pathogenesis. Nef promotes the immune escaping of infected cells. The HIV-1 antisense RNA, *asp* mRNA, is known to suppress viral expression from 5′LTR (as L.545), thus negatively regulating HIV-1 replication, although it has not been reported that *asp* mRNA directly interacts with Tat and Rev. Generated using Servier Medical Art, provided by Servier, licensed under a Creative Commons Attribution 3.0 unported license.

The detailed molecular mechanism of HTLV-1 Rex and HIV-1 Rev in nuclear-export of viral RNA is well-described (77, 85). The importance of HTLV-1 Rex and HIV-1 Rev in the viral replication cycle was elucidated early after the discovery of each virus (75, 76, 86–94). As shown in **Figure 3**, HTLV-1 Rex and HIV-1 Rev share many similarities in the primary structure and the functional mechanism. Rex and Rev are encoded in the 3′ LTR flanking region of HTLV-1 and HIV-1 genome, respectively (**Figure 3A**). They are translated from doubly spliced viral mRNA (**Figure 3B**). Both have NES (the nuclear export signal), NLS (the nuclear localization signal), and two MDs (the multimerization domains) (**Figure 3C**) to regulate viral mRNA transport and the viral particle production through a largely common molecular mechanism (52). Such molecular mechanism of HTLV-1 Rex and HIV-1 Rev was elucidated at a similar rate by the late 1990s. In the 2000s, no further active investigation into HTLV-1 Rex was conducted, while exploration of the more detailed molecular mechanism of HIV-1 Rev was continued (95–98). Ongoing studies to date suggest that HIV-1 Rev is involved not only in viral RNA transport but also in various intracellular pathways such as splicing and translation (99), defining it as the key player in the viral replication cycle and as a potential therapeutic target of HIV-1 (100). In particular, the crystal structure of HIV-1 Rev, which was elucidated around 2010, improved our understanding greatly in the mechanism of Rev/CRM1 interaction *via* multimerization of Rev (101–107). Even now, new virological findings are updated one after another in the virology of HIV-1 including the property of Rev (61, 100, 108–110). In contrast, no new information on the structure of HTLV-1 Rex has been reported since 1999, which demonstrated a partial structure of the N-terminal ARM region and RNA adaptor of Rex (aa1-16) by NMR (111). HTLV-1 Rex has not been analyzed in its involvement in intracellular pathways such as mRNA splicing and translation, either.

### Regulation of viral gene expression during latent infection

Proviral LTRs undergo epigenetic repression in latently infected cells of both HTLV-1 (112–114) and HIV-1 (74, 115, 116). Those epigenetic modifications include chromatin-remodeling and histone modification, as well as DNA methylation. In particular, heavy DNA methylation plays an important role in the repression of viral gene expression, i.e., maintenance of latency.

In HTLV-1 infected cell lines and primary malignant cells from ATL patients, the 5′LTR (as L.545), but not the 3′ LTR, of provirus are highly methylated (117, 118). Recently, a suppressive regulation of HTLV-1 gene expression by CTCF was also reported (119, 120). However, the mechanism of why all HTLV-1-infected cells uniformly switch to full transcriptional latency within a certain period is not known. Taniguchi et al. predicted based on the provirus methylation profiling analysis that the methylation spreads from the proviral gene body to the 5′LTR (as L.545) in carrier infected cells, eventually forming complete methylation of the 5′LTR (as L.545) in ATL cells (118). Experimental infection systems and animal models that can track the transition from early infection to latent infection in a single cell will be necessary to elucidate this mechanism.

The proviral methylation has been also reported in HIV-1 infected cells (121, 122). It has been also reported that the proviral methylation level is not high in many HIV-1 infected individuals, and even fluctuates in an individual over years (123), whole the LTR methylation under ART treatment is rare (124). These observations in primary HIV-1 infected cells indicate that the methylation of LTR and provirus is not the main mechanism for latent persistence in HIV-1 infected T cells.

### The mode of viral spread and pathogenesis

In HTLV-1, the mode of viral spread at the time of transmission has not been fully clarified, although both possibilities of infected T cell transmission and/or *de novo* infection are proposed by *in vitro*/*in vivo* experiments (16). Especially, the transmission of HTLV-1 infected T cells from mother to child through breast milk has been clinically confirmed (125). Most infected cells are expected to be in the transcription latency (deep latency) by heavy methylation of 5′LTR (as L.545) in HTLV-1-infected individuals and patients (117) (**Figure 2A**) since few viral particles are detected peripherally (9). It is also speculated that neither active proliferation of infected T cells nor virion production for *de novo* infection occurs in asymptomatic carriers since the proviral load (ProVL=% proviral DNA carrying T cells in PBMCs) is kept under 4% in most asymptomatic carriers (6). Yet, to maintain a certain level of ProVL, the temporal somatic infected-cell division may occur even in asymptomatic carriers

In HIV-1, after the acute infection phase, the most of infected cells are eliminated except for long-lived memory T cells. The blood viral mRNA level (viral load: VL) in an infected individual is reduced to the set point, yet never be undetectable level without treatment even during chronic infection (126) (**Figure 2B**). This suggests that the survived latent reservoir cells likely to continue producing virions even during the chronic infection phase (115, 126) (**Figure 2B**). It may be because the 5′LTR (as L.545) methylation state of each infected cell is heterogenous in HIV-1 infected individuals (74), thus some infected cells are allowed to continue the leaky expression of viral genes. This hypothesis is also supported by the fact that stopping the ART treatment in HIV-1 carriers restores the viral load quickly in most cases (see reviews (127, 128). These transcriptionally-reversible infected cells (in shallow latency) are reactivated to initiate the viral replication cycle and are clonally expanded by antigen stimulation within a few years. The viral production in an infected cell continues until the host cell dies. Thus, the number of functional CD4^+^ T cells is eventually depleted to develop AIDS (12) (**Figure 2B**).

### *CIS*-acting self-regulatory mechanism of viral gene expression

The above similarities and differences between HTLV-1 and HIV-1 let us speculate that the major factors that distinguish the characteristics of the infected cell and associated disease(s) of these viruses appear to be the regulation and reactivation of viral production in the host cell. As explained above, Tax and Rex for HTLV-1 and Tat and Rev for HIV-1 are essential for viral replication (76). Particularly, HTLV-1 Rex and HIV-1 Rev were shown early on to be essential for viral particle formation *via* export and stabilization of unspliced/singly spliced viral structural mRNAs (129). Therefore, *cis*- and *trans*-regulatory mechanisms of viral proteins, especially of HTLV-1 Rex and HIV-1 Rev, are expected to closely relate to the level of viral production.

In HTLV-1, Rex is thought to regulate the amount of viral particle production, thus determining the timing of active viral replication in the early infection and the entry to the latent state, because the intracellular concentration of structural protein mRNAs is regulated by Rex (51, 130). Rex selectively transports CRM1-dependent viral mRNAs of structural proteins out of the nucleus, thereby reducing the amount of normal cap-dependent mRNA nuclear transport. Although there is a report that some *Tax/Rex* mRNAs are RxRE/CRM1-dependently transported out of the nucleus (131), most are exported cap-dependently, just like other cellular mRNAs. Thus, activation of selective nuclear export of CRM1-dependent viral structural protein mRNAs by Rex reduces the cytoplasmic amount of *Tax/Rex* mRNA to be translated. Therefore, Rex regulates expression levels of Rex itself and Tax in a negative feedback manner (**Figure 3D**). Another viral accessory protein, p30II, which is translated from a minor doubly spliced transcript variant, is known to suppress the viral replication cycle by retention of *Tax/Rex* mRNA in the nucleolus, thus reducing the cellular level of Tax and Rex proteins (34, 132) (**Figure 3D**). P30II is also known to interact directly with Rex to inhibit its activity. The most recent review of p30II (133) highlighted that it is also involved in the host T cell gene expression and cell-cycle regulation, proposing a critical role of this protein in T-cell transformation (**Figure 3D**). In addition, the authors described the important aspect of p30II in the manipulation of innate immune response by suppressing TLR4 (toll-like receptor 4) *via* inhibition of PU.1 transcription factor in myeloid cells, such as macrophages, monocytes, and dendritic cells. P12 is known to activate NFAT and contributes to the proliferation of infected cells (34, 134, 135), although it has not been reported that p12 directly or indirectly influences the activity of Tax and Rex (**Figure 3D**). Finally, Hbz, the only functional viral protein expressed from the antisense strand, suppresses viral gene expression by Tax in early infected cells (**Figure 3D**) and is the only viral factor that continues to be expressed after latency, thus is believed to maintain latently infected cells (136). Recently, Philip et al. reported that Hbz directly suppresses the Rex function in the nuclear export of the RxRE-dependent intron-containing mRNAs (50) (**Figure 3D**). They also demonstrated that Hbz inhibits the function of Rex and Tax in the viral replication cycle in early infection, as well as in reactivation in the latently infected cell. Their data proposed an important notion that Hbz plays a central role in sustaining the latency of HTLV-1 infected cells *via* regulation of Rex and Tax activities (**Figure 3D**). Thus, the viral replication cycle of HTLV-1 is strictly regulated by the *cis*-acting regulatory mechanism among the viral accessory proteins (73, 132).

In HIV-1, the negative-feedback mechanism may be also true between HIV-1 Rev and Tat, as Tat is also encoded in a doubly spliced HIV-1 mRNA (**Figure 3D**). HIV-1 also encodes several accessory proteins such as Nef, Vpu, Vpr, and Vif. They influence the phenotypes and functions of infected CD4^+^ T cells and thus play significant roles in the pathogenesis of HIV-1. On the other hand, it is also known that these accessory proteins are not directly related to viral gene expression and viral replication (137) (**Figure 3D**). HIV-1 encoded antisense RNA, *asp* (antisense protein) RNA, which is the only viral factor to suppress the viral gene expression from 5′LTR (as L.545) *via* epigenetic modification and replication, thus *asp* mRNA is considered to play an important role in the maintenance of the latent infection of HIV-1 (138–141). However, it has not been reported that *asp* mRNA directly interacts with Tat and Rev. The role of ASP protein, which is encoded by a*sp* mRNA, in the viral life cycle has been less clarified compared with the RNA (138, 140). Consequently, it seems that HIV-1 does not have a *cis*-acting regulatory mechanism as strict as HTLV-1, especially in the direct regulation of Tat and Rev activities.

Besides the function of accessory proteins, other factors may be involved in the overall optimization of the power balance among viral proteins in HTLV-1. One of those is that HTLV-1 Rex and Tax are translated from the exact same mRNA, probably allowing the fine-tuning of the expression levels of Tax and Rex by different translation-initiation efficiency of each frame (**Figure 3B**). Moreover, such double-coding mRNA is beneficial to control the expression level of both Tax and Rex simultaneously. As mentioned above, p30II binds to *Tax/Rex* mRNA and confines it in nucleoli, thus restricting the nuclear export and translation of both Tax and Rex together (34, 132). Such a mechanism is unique to HTLV-1. HIV-1 Tat and Rev are encoded on the separated mRNAs without the counterpart of HTLV-1 p30II. In addition, Rex must function longer than Tax in infected cells to serve as the final regulator to shut down the viral replication and to transit to the latency. Indeed, as shown in **Figure 3C**, HTLV-1 Rex has the C-terminal stability domain (142), which is responsible for the significantly longer half-life of Rex than Tax (51). No clear stability domain has been reported for HIV-1 Rev (**Figure 3C**). Recently, a linker region at both ends of the N/C terminus of Rev was shown to be involved in stability (108), but the biological importance of the stabilization in the regulation of Rev function and for Tat activity are still unknown.

**Figure 4:**
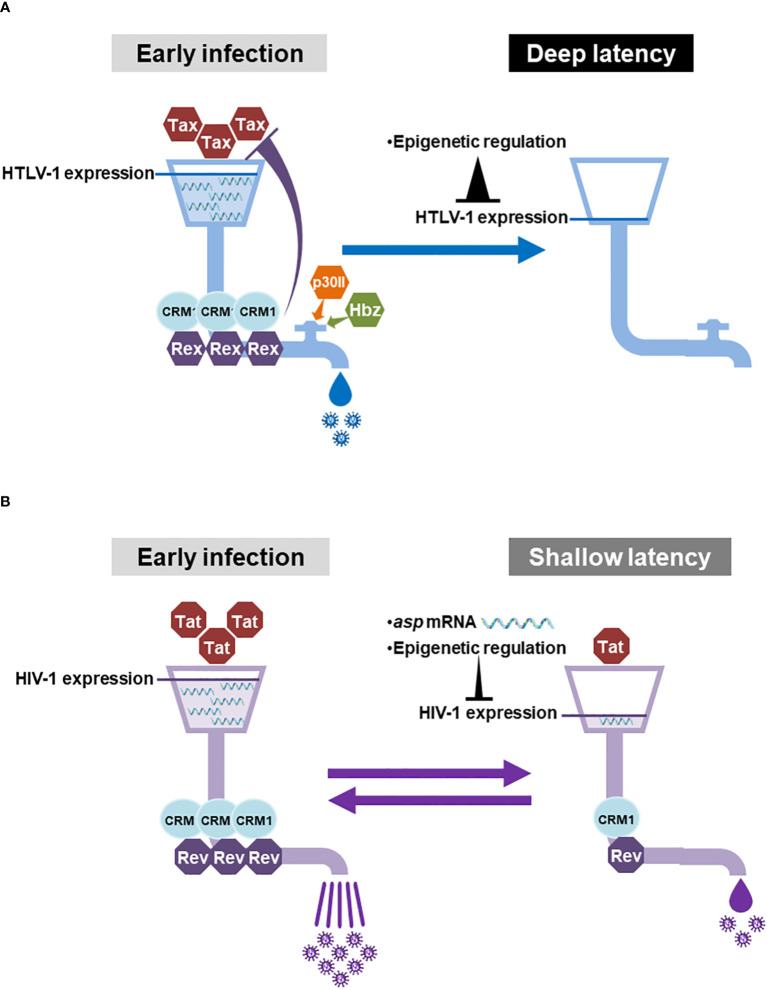
With or without the regulatory valve for HTLV-1-Rex and HIV-1 Rev and the regulation of the viral gene expression: **(A)** In the early HTLV-1-infected cells, Tax enhances the viral gene expression and Rex serves the “pipeline” of viral mRNAs between the site of transcription and the translational machinery. Since the “flow rate (Rex activity)” of the Rex pipeline is controlled by the “regulatory valve”, which is composed of Rex itself, Hbz, and p30II, the viral mRNAs transcribed by Tax are not all translated directly into viral particles. In the latent state, the 5′LTR (as L.545) is heavily methylated universally, therefore the expression of Tax and Rex is completely shut-down in almost all infected T cells. Such strict regulation of viral gene expression may allow the infected cells sustain the disease-free deep latency for decades. **(B)** In HIV-1 infected cells, Tat is responsible for viral gene expression and Rev also serves as the pipeline of the viral mRNAs. In contrast to HTLV-1, there is no regulatory valve, which controls the activity of Rev. Therefore, the viral mRNAs transcribed by Tat are expected to be translated as is to produce a vast number of viral particles from an early infected cell. *Asp* mRNA and methylation of 5′LTR (as L.545) serve to suppress viral gene expression during the latency. However, the methylation in the 5′LTR (as L.545) does not occur universally, allowing some reservoir cells to produce a low level of viral particles even in latently infected cells. The lack of a regulatory valve on the Rev pipeline together with a permissive viral gene expression may allow the infected cell to stay in the shallow latency, which is easily reactivated. Generated using Servier Medical Art, provided by Servier, licensed under a Creative Commons Attribution 3.0 unported license.

Collectively, HTLV-1 has multiple factors, which negatively regulate the activity of Rex and Tax, while HIV-1 has a less-stringent regulation against HIV-1 Rev and Tat activities (**Figure 3D**). Since HTLV-1 Rex and HIV-1 Rev serve as the “pipeline” of the viral transcripts for replication, the regulation of the “flow rate (i.e., the activity of Rex and Rev)” is critical for the overall viral production. We imagine that the Rex pipeline has a “regulatory valve” consisting of multiple *cis*-acting regulators such as Hbz and p30II (**Figure 4A**). Therefore, the viral mRNA transcribed by Tax is not translated as is, but tuned by the regulatory valve, which controls the Rex activity. The regulatory valve on the Rex pipeline thereby controls the active viral replication phase in early HTLV-1 infection and rapid replication cessation to enter the latency. In addition to such orchestrated mechanism in the quantity and temporal regulation of the viral replication cycle, the universal 5′LTR (as L.545) methylation stops Tax and Rex expression completely, thus allowing the infected cell to maintain the deep latency thereafter (**Figure 4A**). In HIV-1, no direct negative regulator of Rev has been reported, at least to date. Since the Rev-pipeline does not have any regulatory valve, the viral transcripts are expected to be translated as are transcribed by Tat (**Figure 4B**). The *asp* mRNA is known to suppress the viral gene expression in early and latent infection. However, since the 5′LTR (as L.545) LTR of HIV-1 provirus is not methylated universally, a leaky viral expression continues at a low level in latency. No regulatory valve on the Rev pipeline and leaky suppression of viral gene expression may be related to the shallow latency of HIV-1 infected cells, which are easily reactivated (**Figure 4B**).

## Reaffirming Rex as a key regulator of viral gene expression

**Figure 5:**
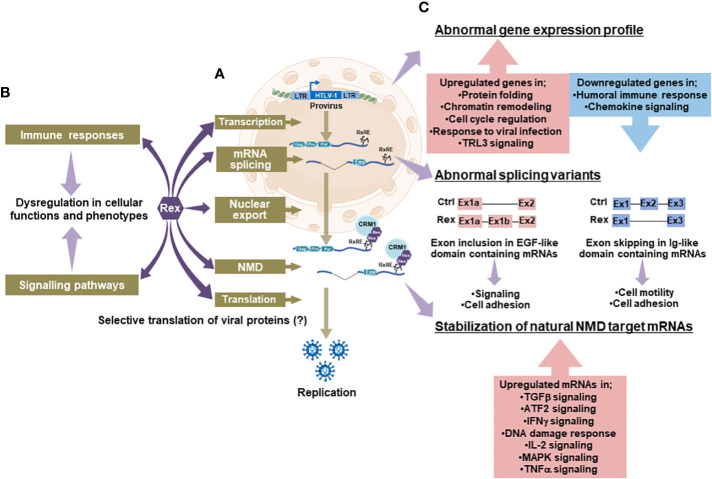
Novel aspects of Rex and its effects on cellular pathways; **(A)**The interactome analysis between Rex and human cellular proteins revealed that Rex interacts with proteins involved in cellular pathways from transcription to translation (arrows in deep purple). Especially, Rex shows interactions with many ribosomal proteins, indicating a possible involvement of Rex in the host translational pathway. **(B)** Rex also interacts with proteins involved in immune response and signaling pathways (arrows in deep purple), suggesting that Rex may alter T cell functions and phenotypes by affecting these intracellular pathways (arrows in light purple). **(C)** Rex-overexpressing T cells showed alterations in a wide range of gene expression profiles (arrows in light purple). In the Rex expressing cells, the genes involved in the regulation of gene expression, cell cycle, and viral infection response are upregulated, while those involved in the humoral immune response and chemokine signaling pathway are downregulated. In addition, changes in splicing patterns were observed in various mRNAs in Rex expressing T cells (arrows in light purple). Particularly, exon inclusion in mRNAs encoding EGF-like domains and exon skipping in mRNAs encoding Ig-like domains are observed in high frequencies. In addition, the expression levels of natural NMD target mRNAs involved in various signaling pathways were elevated in Rex expressing T cells (arrows in light purple), possibly because of the NMD inhibitory function of Rex. Generated using Servier Medical Art, provided by Servier, licensed under a Creative Commons Attribution 3.0 unported license.

The RxRE/CRM1 dependent unspliced/singly spliced viral mRNA nuclear export by HTLV-1 Rex has been well studied. However, how Rex achieves such a function is largely unknown. Especially, the following questions have not been clarified, yet; 1) How does Rex avoid unnecessary splicing?, 2) Why does Rex preferentially use CRM1, a major nuclear export adapter of the host cell?, and 3) Does the RxRE/CRM1 dependent pathway contribute to the selective translation of viral RNAs? We speculate that Rex has as yet unknown aspects to escort viral RNAs from the transcription to the translation. To explore new aspects of Rex, we recently conducted transcriptome and interactome analysis of Rex (143). Firstly, we conducted the interactome analysis of Rex in HEK293T cells overexpressing His-Halo-tagged Rex. The human intracellular proteins that interact with Rex were identified by the His-tag/Halo-tag tandem affinity purification followed by the LC-MS/MS analysis. The results revealed that Rex interacts with human intracellular proteins involved in various pathways such as gene expression, splicing, mRNA quality control, and translation (**Figure 5A**). In particular, Rex interacts with a large number of ribosomal proteins, which highlighted the involvement of Rex in the host cellular translational pathway. How Rex intervenes in the host translation to attain the selective translation of viral mRNAs *via* the RxRE/CRM1 dependent mechanism waits for further investigation. Rex also interacts with proteins involved in immune responses and signaling pathways (**Figure 5B**), proposing a possibility that Rex may alter the phenotype of the host T cell. Next, we conducted the gene expression and splicing analysis of Rex in CEM (human TALL patient-derived T cell line) overexpressing Rex. The Rex-overexpressing induced alterations in a wide range of gene expression profiles (**Figure 5C**). The significantly upregulated genes by Rex are involved in the regulation of gene expression, cell cycle, and viral infection response. The significantly downregulated genes by Rex are involved in the humoral immune response and chemokine signaling pathway. The exon microarray analysis shows that the mRNA splicing patterns of more than 2,000 mRNAs are altered in the Rex-overexpressing CEM cells. These results indicate that Rex influences the host cellular gene expression and splicing mechanism. Changes in gene expression levels and the splicing patterns by Rex may influence the protein activities and functions and thus may re-shape the host cellular function. Previously, we reported that Rex suppresses the host cellular nonsense-mediated mRNA decay (NMD) to stabilize the viral genomic RNAs (53). Recently, we analyzed the interaction between Rex and the NMD complex proteins and demonstrated that Rex may enter the NMD complex from the beginning to the end of the pathway to regulate the activity of NMD (54). Indeed, our transcriptome analysis in Rex overexpressing CEM demonstrated the upregulation of natural NMD target mRNAs, which are involved in TGFβ, ATF2, IFNγ, DNA damage response, IL-2, MAPK, and TNFα signaling pathways. Therefore, Rex may influence the activities of various signaling pathways *via* the suppression of NMD. These results suggest that Rex not only transports viral RNA outside the nucleus but may also intervene in various host intracellular pathways (**Figure 5**). To transport viral RNAs, Rex needs to avoid extra splicing at the site of transcription and protect them from NMD, which targets and degrades abnormal mRNAs including intron-containing viral mRNAs at the site of translation. Based on our data, we hypothesize Rex escorts viral unspliced/singly spliced mRNAs by regulating the activity of the various intracellular pathways through which the mRNAs are processed. **Figure 5** may reflect such novel aspects of Rex and lets us reaffirm Rex as a key viral factor in viral gene expression.

## Conclusion remarks and future perspectives

HTLV-1 Rex and HIV-1 Rev have long been thought to positively-regulate viral gene expression *via* the transport of viral RNA. In this review, we note that HTLV-1- and HIV-1-infected T cells have different characteristics, despite the many similarities in the functional mechanisms of these accessory proteins. By exploring the similarities and differences between HTLV-1 and HIV-1, we proposed a novel possibility that the presence or absence of a virus-derived regulatory valve for Rex or Rev activity, respectively, may relate to the permissiveness of the viral gene expression, thus the phenotype and pathogenesis of the infected cell. Our recent study, which demonstrated the involvement of HTLV-1 Rex in a much wider range of host-cellular pathways than ever expected (**Figure 5**), supports the notion that ruling viral gene expression at the Rex activity may be beneficial for the virus to control the overall events in the infected cell, i.e., early viral particle replication and maintenance of stable transcriptional latency thereafter. In HTLV-1,not only Tax but also Hbz directly influences the viral gene expression. They are also known as the oncoproteins of HTLV-1, which drive the transformation of the infected T cell. Especially, Hbz is known to be expressed in all infected cells through the lifetime of an infected individual even during the latent infection in an asymptomatic carrier. As shown in **Figure 1**, it is also well-known that HTLV-1-infected cells are not transformed in about 95% of infected individuals. Consequently, we speculate that there must be a viral mechanism to suppress the transforming activity of Tax and Hbz. Especially, the function and activity of Hbz in the latently infected T cells need to be controlled, otherwise, all HTLV-1 infected cells eventually would be malignantly-transformed to develop ATL in all infected individuals. Recently, we tested the effects of HTLV-1 Rex, Tax, and Hbz in CEM cells expressing them individually or simultaneously. As result, the intracellular changes observed by Rex, Tax, or Hbz alone are significantly different from those by Rex/Tax, Tax/Hbz, Rex/Hbz, and Rex/Tax/Hbz together. (Nakano, unpublished data). These results propose a possibility that those HTLV-1 accessory proteins regulate each other. Such inter-control within a viral replication cycle may be essential to orchestrate the events in the HTLV-1 infected T cells, i.e., the early replication phase followed by stable maintenance of a disease-free latent state. We believe that the further investigation in the functional interaction among HTLV-1 accessory proteins will bring us closer to understanding the nature of the HTLV-1 virus and its infected cells in the future.

## Author contributions

KN wrote the article and TW reviewed it. Both KN and TW supervised and conducted experiments in functional analysis of HTLV-1 Rex. All authors contributed to the article and approved the submitted version.

## Funding

This work was supported by Grants-in-Aid for Scientific Research from the Ministry of Education, Culture, Sports, Science, and Technology of Japan, (15K06827) and (19K07573) to KN, and (20KK0181) to TW.

## Acknowledgments

We thank very much all the corroborators and students in the Department of Computational Biology and Medical Scienced, Graduate School of Frontier Sciences, The University of Tokyo, for their participation and assistance in the research of HTLV-1 Rex.

## Conflict of interest

The authors declare that the research was conducted in the absence of any commercial or financial relationships that could be construed as a potential conflict of interest.

## Publisher’s note

All claims expressed in this article are solely those of the authors and do not necessarily represent those of their affiliated organizations, or those of the publisher, the editors and the reviewers. Any product that may be evaluated in this article, or claim that may be made by its manufacturer, is not guaranteed or endorsed by the publisher.
